# Polyurethane/Vermiculite Foam Composite as Sustainable Material for Vertical Flame Retardant

**DOI:** 10.3390/polym14183777

**Published:** 2022-09-09

**Authors:** Lívia R. P. Silva Tenório Alves, Márcio Davi Tenório C. Alves, Luzia M. Castro Honorio, Alan I. Moraes, Edson C. Silva-Filho, Ramón Peña-Garcia, Marcelo B. Furtini, Durcilene A. da Silva, Josy A. Osajima

**Affiliations:** 1 Limav, Interdisciplinary Laboratory for Advanced Materials—LIMAV, UFPI, Teresina 64049-550, PI, Brazil; 2 Department of Mechanical Engineering, Technology Center, UFPI, Teresina 64049-550, PI, Brazil; 3 Department of Chemistry and Physics, Center for Agrarian Sciences, UFPB, Areia 58397-000, PB, Brazil; 4 Federal Rural University of Pernambuco, Unidade Acadêmica do Cabo de Santo Agostinho, Cabo de Santo Agostinho 54518-430, PE, Brazil; 5 Research Center on Biodiversity and Biotechnology, BIOTEC, Parnaíba Delta Federal University, UFDPar, Parnaíba 64202-020, PI, Brazil

**Keywords:** polyurethane foams, polymer–clay composites, flame retardant

## Abstract

Rigid polyurethane foams were prepared by the one-step expandable foam method using casting molding followed by forming clay-based composites. Polyurethane/vermiculite foam composites (PU/VMT) were controlled based on adding the percentage of clay in the formulation. The effects of composite modifications were evaluated by X-ray diffraction (XRD), thermogravimetric analysis (TG/DTG), and scanning electron microscopy (SEM/EDS) applied to the flame retardancy explored by the vertical burn test. The results indicated that adding clay controlled the particle size concerning polyurethane (PU) foams. However, they exhibited spherical structures with closed cells with relatively uniform distribution. XRD analysis showed the peaks defined at 2θ = 18° and 2θ = 73° relative to the crystallinity in formation and interaction of rigid segments were identified, as well as the influence of crystallinity reduction in composites. In the flame test, the flame retardant surface was successful in all composites, given the success of the dispersibility and planar orientation of the clay layers and the existence of an ideal content of vermiculite (VMT) incorporated in the foam matrix.

## 1. Introduction

Polyurethane foams (PU) without flame retardant additives are commercial materials with low thermal conductivity, low density, and low cost [1,2]. However, they have a flammable character and rapid flame propagation through the formation of a fire pool under the burning object [3]. Therefore, they are widely used as thermal insulators in both construction and engineering applications and the development of heat transfer technologies, making them an attractive resource for minimizing energy waste caused by heat transfer [4,5].

Thermal insulation deserves special attention in the development of heat transfer technology [4]. However, despite the many advantages of PU, many researchers often seek optimization in flame retardancy and smoke suppression. Flame retardant PUs can be produced by halogenated compounds [6], expanded graphite (EG) [7], ammonium polyphosphate (APP) [8], melamine derivatives [9], microencapsulated red phosphorus, and others [10]. Halogenated compounds have been banned by several countries, such as France [11], the United States [12], and the European Union [13], due to their toxicity and bioaccumulation. On the other hand, other flame retardant additives always harm PU, which can damage PU's mechanical and thermal insulation properties, causing the leaching of flame retardants [14].

In recent years, polyurethane composites and/or polymeric/clay materials have gained special attention from industries since the combination has excellent thermo-mechanical properties and a series of physical and chemical properties [15]. These polymer-clay composites can be synthesized by incorporating inorganic clays dispersed in a polymer matrix. The easy availability, sustainable properties, and low cost of clay minerals require a small weight percentage of the clay to produce a helpful nanocomposite, with a remarkable improvement in properties. Such as tensile strength and modulus, gas permeability, heat distortion, temperature, and flammability without affecting the optical homogeneity of the products make these fillers attractive [16,17].

The layered clays’ low percentage (≤10% by weight) for the PU matrix can significantly improve many essential properties, such as tensile strength, modulus, and elongation at break, tear strength, and flame retardant properties. Polymer/ceramic materials generally exhibit low thermal conductivity and are widely used as thermal insulators. The thermal properties of these elements can be optimized by incorporating tiny pores, which are created by foaming throughout the production method [18].

Among the clays used for the preparation of polymer–clay nanocomposites [19,20], there is vermiculite (VMT), which is a clay mineral with a basic structure composed of thin sheets of crystals, usually bonded face to face, conceiving a unit cell of two tetrahedral sheets separated by an octahedral. Vermiculite is a swelling hydrous phyllosilicate clay mineral with an ideal chemical formula of (Mg^2+^, Fe^2+^, Fe^3+^)3[(SiAl)_4_O_10_]OH_2_⋅4H_2_O [21]. As vermiculite can be expanded up to 30 times its original volume when heated to 650–950 °C, expanded vermiculite (EV) is often used as an additive in materials and geopolymers to obtain greater porosity and fire resistance [22,23,24,25].

Vermiculite is a swelling hydrous phyllosilicate clay mineral with an ideal chemical formula of (Mg^2+^, Fe^2+^, Fe^3+^)_3_[(SiAl)_4_O_10_]OH_2_⋅4H_2_O [21]. Vermiculite is among the most commonly used silicates in the manufacture of bio-nanocomposites. Mineral clays can modify the polymer’s characteristics and improve its processability. These silicates are composed of tetrahedral (silicon, tetracoordinate) and octahedral (aluminum, hexacoordinated) layers with a lamellar crystallinity structure that is formed by a 2:1 mineral clay unit cell [26,27,28].

The objective of this work was to investigate the morphological, structural, and thermal stability properties of rigid foams of the PU/VMT composite in different concentrations (0, 5%, 10%, 15%, and 20%), as well as contribute to the development of environmentally friendly flame retardant products and sustainable growth.

## 2. Experimental

### 2.1. Reagents

Gaya In purchased medium-expanded vermiculite. Minerals Industries. The polyol used in the PU/VMT is polypropylene glycol with a viscosity at 20 °C = 1500 ± 300 cps (MCNS Inc., Seoul, Korea) containing n-pentane (technical grade). The isocyanate used was 4,4′-diphenylmethane diisocyanate (MDI) with NCO% = 31% and an average functionality of 2.8 (Tosoh Corporation, Tokyo, Japan). The reagents were used without purification.

### 2.2. Preparation of Rigid Foam and Composites

**Figure 1:**
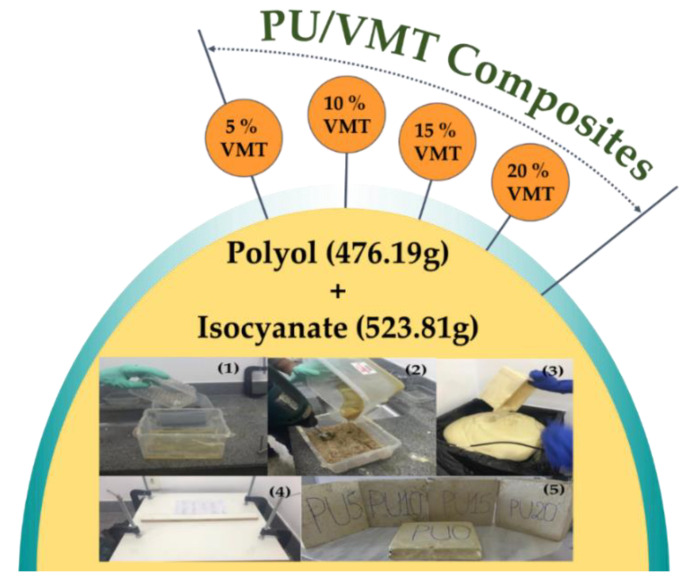
Stages of preparation of rigid polyurethane foams. Stages of preparation of rigid polyurethane foams. (**1**) Vermiculite (VMT) dispersed in the polyol before adding the isocyanate; (**2**) MDI isocyanate was added to the mixture; (**3**) The mixture was then quickly poured into a mold to produce PU foam; (**4**) The molds containing PU were kept at room temperature for 24 h (**5**) PU foams.

Rigid polyurethane foams were prepared using the one-step expandable foam method with casting molding. First, vermiculite (VMT) was dispersed in the polyol before adding the isocyanate (Figure 1 (1)). Then, a certain amount of MDI isocyanate (MDI/polyol = 1.1 *w*/*w*) was added to the mixture with vigorous stirring for 10 s (Figure 1 (2)). The mixture was then quickly poured into a mold to produce PU foam (Figure 1 (3)). The molds containing PU were kept at room temperature for 24 h (Figure 1 (4)). Finally, the PU foams were separated from the mold, and the hard surface of the foams was removed (Figure 1 (5)). Ten samples of each formulation were prepared. The composites were called the PU/VMT x%, where x is the amount of clay used. The foam components are shown in Table 1.

### 2.3. Characterizations

The PU/VMT composites were characterized by Scanning Electron Microscopy (SEM) using a scanning electron microscope with an electron source by field emission FEG (Field Emission Gun), Quanta FEI 250. The micrographs were obtained at 20 kV and a spot size of 3 with a Dispersive Energy Spectrometer (EDS), making it possible to evaluate their chemical composition semi-quantitatively. In addition, thermal gravimetric analysis was performed on a Shimadzu T.G., model TGA-51, under air with a heating rate of 10 °C·min⁻^1^ from room temperature to 1000 °C.

X-ray diffractometer (XRD) analysis was performed on the composites in a Panalytical X-ray diffractometer, Model Serie Empyrean. First, the crystallinity index of each sample was determined by the peak intensity method represented by Equation (1).

IC = (Ic − Ia)/Ic × 100%(1)

where:

IC is the crystallinity index (%);

Ic is the intensity of the crystalline peak (a.u.);

Ia is the intensity of the amorphous peak (a.u.).

### 2.4. Vertical Flammability Test

The flammability tests performed vertical tests according to the standard vertical burning test (ASTMD 3801-96) [29]. The sample size was 130 × 13 × 3 mm (length × width × thickness).

According to the ASTMD 3801-96 Vertical Standard Burn Test (Standard Test Method for Measuring the Comparative Burning Characteristics of Solid Plastics in a Vertical Position), we calculate the total after-flame time for each set of five specimens, *t_f_*, using the following Equation (2):

(2) $$t\left(f\right)=\sum_{i=1}^{5}\left(t_{1,i}+t_{2,i}\right)$$

where:

*tf* = total flame time, s;

*t*_1_,*_i_* = after-flame time after the first flame impact, s;

*t*_2_, *_i_* = after-flame time after the impact of the second flame, s, of the i-specimen.

For the vertical Standard Burn Test (ASTMD 3801-96) [29], five tested samples of each formulation were tested. The samples were tested in a room atmosphere and 45 to 75% relative humidity.

## 3. Results and Discussion

### 3.1. Analysis of TG/DTG, DRX, MEV, and EDS

**Figure 2:**
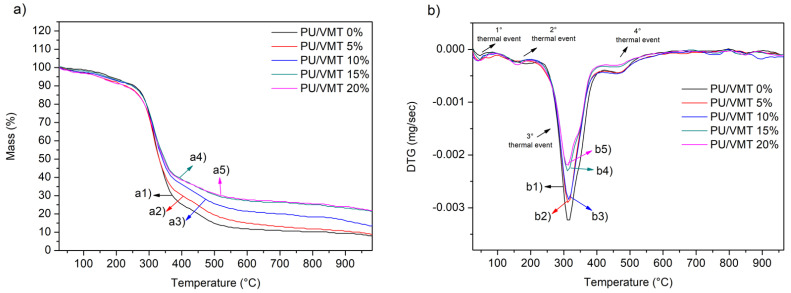
(**a**) TG curves of the composites: PU/VMT 0% (a1), PU/VMT 5% (a2), PU/VMT 10% (a3), PU/VMT 15% (a4) and (**b**) DTG: PU/VMT 20% (a5); DTG: PU/VMT 0% (b1), PU/VMT 5% (b2), PU/VMT 10% (b3), PU/VMT 15% (b4), and PU/VMT 20% (b5).

The thermal stabilities of the PU/VMT composites x% (x% = 0%, 5%, 10%, 15%, and 20%) were studied and analyzed by the behavior of the TG/DTG curves shown in Figure 2 with the thermal and in Table 2. For the control material, PU/VMT 0% (Figure 2, (a1 and b1)), it was possible to observe four stages of mass losses, the first referring to the loss of water or physisorbed gases. The other losses are characteristics of the PU occurring in the second event, including the decomposition of the carbamate group in the main chain of the PU in the C–O bond to form isocyanate and polyol. In the third event, the continuation of the decomposition of intermediates (diphenyl ethyl allophanate) occurs in primary or secondary amines, alkene, and CO_2_. In the fourth event, polyol chains are degraded, releasing a large amount of CO_2_. The values of the thermal events are summarized in Table 2 [30,31,32,33]. The materials PU/VMT 5% (Figure 2 (a2 and b2)), PU/VMT 10% (Figure 2 (a3 and b3)), PU/VMT 15% (Figure 2 (a4 and b4)), and PU/VMT 20% (Figure 2 (a5 and b5)) showed a profile similar to the control material PU/VMT0%, with the exception of the residue value. This phenomenon is similar to the results of Meng et al. [31]. They studied a self-healing coating based on flexible polyurethane foam, and the work of Wang et al. [9] presents very similar profiles of the TG and DTG curves of PU foams. Furthermore, Miedzińska et al. [34] indicate stages of thermal decomposition around Tmax = 215 °C (1st Stage), 319 °C (2nd Stage), and 591 °C (3rd Stage), corroborating the data obtained in this work experimentally.

For the 0% PU/VMT control, there is an amount of mass residue of approximately 9.095% (Table 2). However, after the addition of clay, there is an increase in the residual mass, which increases as the clay increases in the material composition: PU/VMT 5%; PU/VMT 10%; PU/VMT 15%; and PU/VMT 20%, 9.93%, 13.72%, 22.53%, and 23.53% (Table 2), respectively, showing a relationship between the degradation events (mainly from a temperature above 300 °C, the increase in the amount of clay, and residual masses. This residual mass increases as clay do not decompose equally to PU (organic compound), as it is a mineral. Most of its mass does not degrade with temperature, with only water leaving the condensation of hydroxyl groups on the surface and structure of the VMT. Curve 2 (b) shows the derivatives of the TG curve. It is possible to observe the similarity of events in all systems, with a significant variation occurring in the third event, with maximum degradation at 310 °C. The highest intensity (or area) occurred for PU/VMT0% until the worst occurred for PU/VMT20%. It corroborates with the previous discussion since the integration of the area of this peak is proportional to the degraded mass in the temperature range, indicating once again the presence of clay and the proportionality with the mass added in different systems.

**Figure 3:**
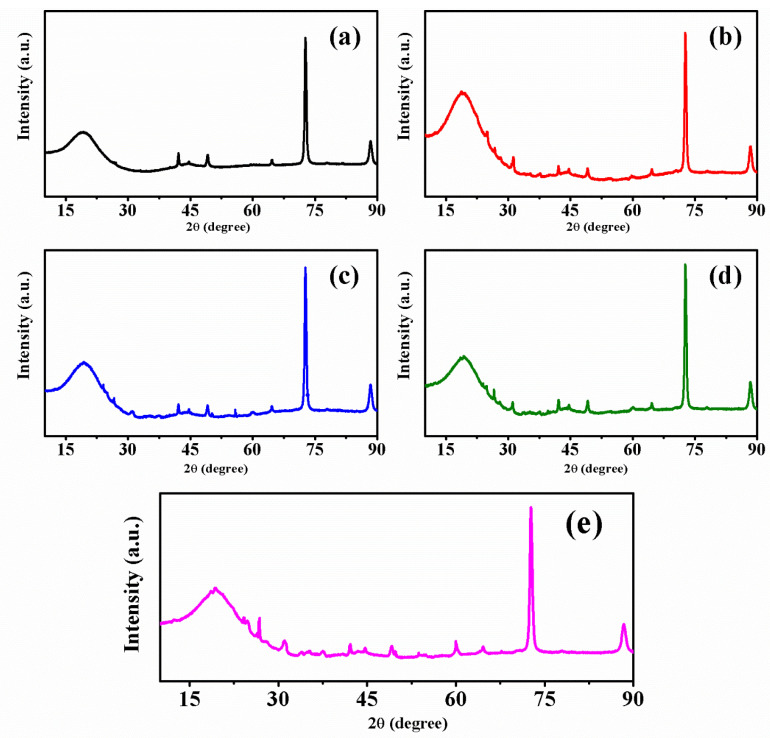
Composite diffractogram: PU/VMT 0% (**a**), PU/VMT 5% (**b**), PU/VMT 10% (**c**), PU/VMT 15% (**d**), and PU/VMT 20% (**e**).

Figure 3 shows the diffractograms of the PU/VMT composites (0%, 10%, and 20%) with a broad peak at 2θ = 18°. Another is defined at a 2θ = 73° relative to the crystallinity in the formation and interaction of rigid segments. The diffractograms show the predominantly amorphous nature of polyurethane and its derivatives, given the absence of narrow diffraction peaks [35].

The crystallinity reduces with the increase of vermiculite up to a limit of 20% m/m of vegetal load. Using the peak intensity measurement method, it is possible to identify that the crystallinity index for polyurethane and its composites was approximately 82.19% for PU/VMT 0%, 64.58% for PU/VMT 5%, 72.61% for PU/VMT 10%, 74.38% for PU/VMT 15%, and 63.78% for PU/VMT 20%. Therefore, in all compositions, the addition of vermiculite influenced the formation and compaction of the rigid segments, which explains the reduction of crystallinity in the composites (Figure 3).

**Figure 4:**
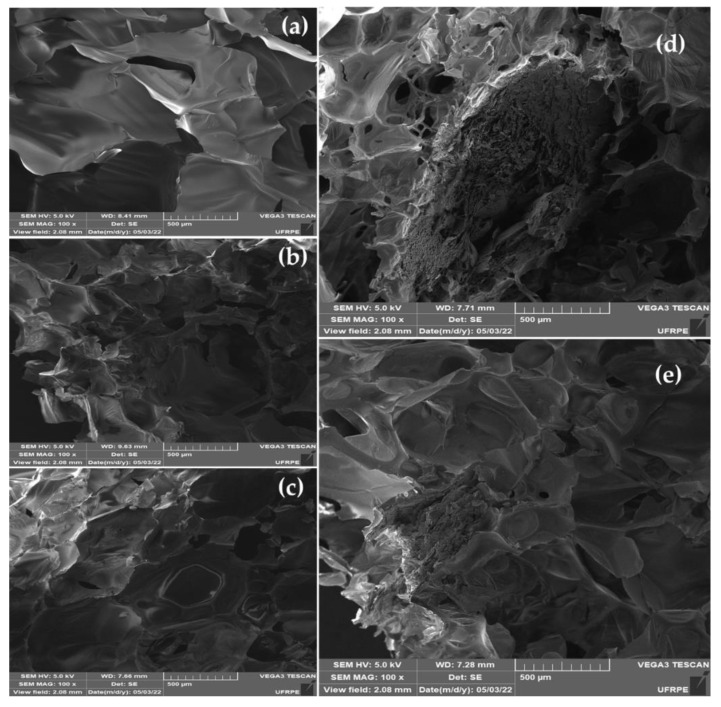
SEM micrographs of the composites: PU/VMT 0% (**a**), PU/VMT 5%(**b**), PU/VMT 10% (**c**), PU/VMT 15% (**d**), and PU/VMT 20% (**e**).

Figure 4 shows SEM/EDS images of the surfaces of PU/VMT composites. All microscopy exhibits closed spherical structures and energetically stable faces. In addition to presenting nucleation sites not regularly distributed due to agglomeration and/or excess VMT charges, resulting in the formation of non-uniform morphology, as shown in Figure 4. For Xia et al. [36], this phenomenon is explained by the fact that EV acts as an inorganic filler on the composite wall, restricting the increase in size, distribution, and uniformity. For the pure composite, it is possible to observe a larger size of disordered structures about the composites with 5%, 10%, 15%, and 20% of organic clay. This same behavior was observed by Harikrishnan et al. [37] when evaluating micrographs of precursor polyurethane foams with a crosslinked glassy carbon. For the authors, the cell size decrease of carbon foam made of composite foam precursor is clear about carbon foam made of pure PU foam.

The incorporation of vermiculite (5%, 10%, 15%, and 20%) interferes with the diameter and distribution of pores due to the increase in the viscosity of the polyol [30], suggesting that the amount of clay changes the orientation and dispersion with to the foaming process since it can act as a nucleating agent due to its good compatibility with polyether and consequently standardize the nucleation sites [38]; that is, the nucleation energy barrier favors the charge–polymer interface [3]. Wang et al. [30] reinforce the relationship between the actual identification of the clay. The surroundings of the core in the form are generated by interface polymer and clay dispersed in the medium. The morphology and properties of composites depend on several factors that drive the distribution and relative percentages, such as the functional groups of the incorporated clay, the synthesis procedure, the molecular weight of the polyols, chemical reactions, and the physical interactions involved [39].

**Figure 5:**
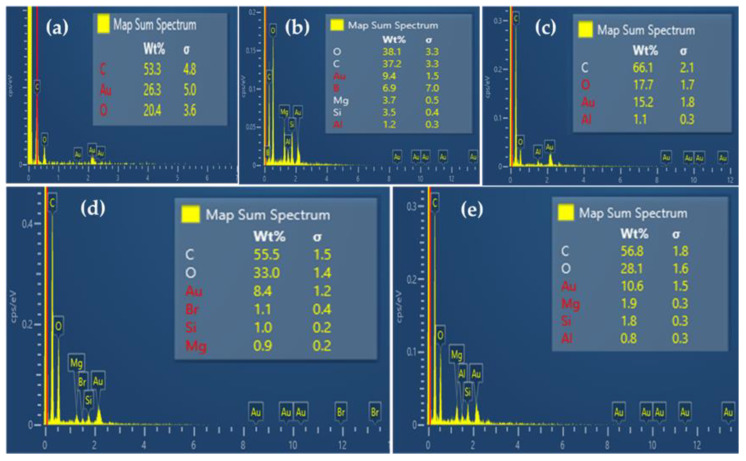
EDS profile of composites: PU/VMT 0% (**a**), PU/VMT 5% (**b**), PU/VMT 10% (**c**), PU/VMT 15% (**d**), and PU/VMT 20% (**e**).

The EDS analysis (Figure 5) shows the elemental composition of the PU/VMT composites. The composite without clay in the PU precursor foam presents the typical elements, such as carbon (53.3%), presence of oxygen (20.4%), and gold (26.3%) attributed to the sample holder of the equipment. The other composites in the presence of vermiculite present boron, aluminum, silicon, and magnesium, arising from the decomposition of clay, which is an aluminosilicate composed of numerous chemical elements, thus suggesting the incorporation of clay into PU. In small amounts, chlorine (PU/VMT 5%) and bromine (PU/VMT 10%) are residual elements.

### 3.2. Performance of Composites in Vertical Flame Test

**Figure 6:**
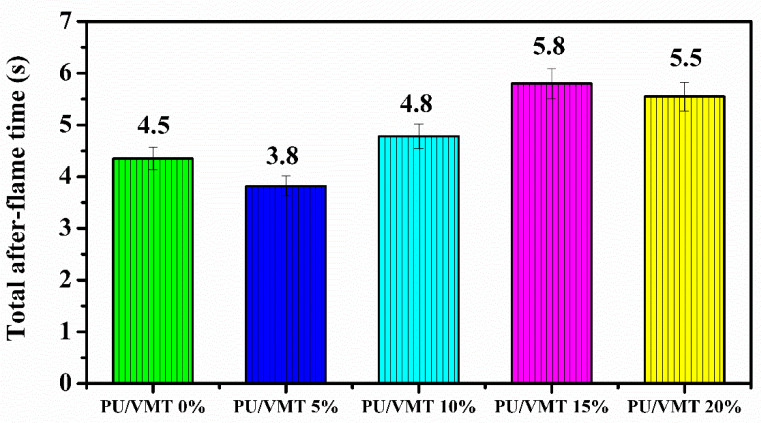
Flammability tests vertical.

The vertical flame test is a quick and direct way to evaluate flame retardancy [40]. In the vertical flammability test (Figure 6), the total post-flame time was observed for each set of composite compositions (0%, 5%, 10%, 15%, and 20%). VMT acts as a reinforcing filler, and as it is an inorganic material, it can contribute to reducing the flammability of polymeric materials. The flame resistance is vertical, and the composite containing 15% VMT showed better firing performance. In addition, it achieved better dispersibility and planar orientation of the clay layers, giving an ideal VMT content to be incorporated into the foam matrix.

The proportion of the mixture between the components guarantees high activity in the interaction between the phases (PU + VMT) and a satisfactory performance [36,41]. In addition, VMT is natural and economical clay, widely used mainly because it is thermostable and flame retardant, which may have favored the flame result [34,42], as shown in Figure 6. However, the flame test of the PU foams did not show regularity with the increase of vermiculite from 0 to 20%. It is known that when exposed to fire, the PU layers start to burn quickly and form an inflatable layer of carbon [43]. We believe that after incorporation with VMT, the exposed area of the foams decreases, hindering the ventilation of oxygen and consequently the burning [43]. Thus, it is inferred that at that moment, the PU mass ratio per unit volume was reduced with the increase of VMT incorporation. In turn, it needs more oxygen to maintain the burning after adding clay [43]. In addition, another point to note is that excess VMT prevents homogenization in the matrix, which would result in the formation of an uneven residual carbon layer after firing, leading to decreased firing when the VMT content exceeds 15%.

## 4. Conclusions

PU/VMT composites were successfully synthesized by the casting molding method. The mixture of vermiculite with PU provided good dispersion and proportionality, given the added compositions of the clay. The PU/VMT15% composite is uniform, reinforcing the cost-benefit issue with environmental and economic advantages. This behavior reinforces the flammability data, and the addition of VMT affected the formation and compaction of the rigid segments, reducing the degree of crystallinity of the composites. The flammability was reduced in PU/VMT 15% composite, showing better burn performance and consequently achieving better dispersibility and planar orientation of the VMT layers.

## Figures and Tables

**Table 1:** PU/VMT composite formulations.

Samples	Polyol	Isocyanate	VMT
PU/VMT 0	476.19 g	523.81 g	-
PU/VMT 5	476.19 g	523.81 g	5%
PU/VMT 10	476.19 g	523.81 g	10%
PU/VMT 15	476.19 g	523.81 g	15%
PU/VMT 20	476.19 g	523.81 g	20%

**Table 2:** Weight loss, temperature range, maximum degradation temperature, and residue of samples.

Sample	Weight Loss (%)	Temperature Range (°C)	Maximum Degradation Temperature (°C)	The Residue (%)
PU/VMT0%	1.07%	25–86 °C	47 °C	9.10%
	6.83%	86–225 °C	182 °C	
	69.34%	225–425 °C	310 °C	
	14.77%	425–985 °C	468 °C	
PU/VMT5%	3.02%	25–99 °C	47 °C	9.93%
	4.42%	99–188 °C	157 °C	
	64.76%	188–422 °C	312 °C	
	17.88%	422–988 °C	459 °C	
PU/VMT10%	3.02%	21–96 °C	41 °C	13.72%
	4.42%	96–196 °C	153 °C	
	64.76%	196–417 °C	314 °C	
	17.88%	417–982 °C	461 °C	
PU/VMT15%	3.02%	24–85 °C	46 °C	22.53%
	4.42%	85–195 °C	164 °C	
	64.76%	195–421 °C	311 °C	
	17.88%	421–987 °C	458 °C	
PU/VMT20%	3.02%	28–86 °C	40 °C	23.53%
	4.42%	86–197 °C	160 °C	
	64.76%	197–426 °C	314 °C	
	17.88%	426–983 °C	454 °C	

## Data Availability

Not applicable.
